# Thermal performance, entropy generation, and machine learning insights of Al₂O₃-TiO₂ hybrid nanofluids in turbulent flow

**DOI:** 10.1038/s41598-025-93749-0

**Published:** 2025-05-08

**Authors:** Praveen Kumar Kanti, V. Vicki Wanatasanappan, Nejla Mahjoub Said, Suman Saini, Vijayalaxmi Mishra, Prabhu Paramasivam, Mohamed Yusuf

**Affiliations:** 1 Institute of Power Engineering, Universiti Tenaga Nasional, Jalan IKRAM-UNITEN, 43000 Selangor, Malaysia; 2 Department of Physics, College of Science, King Khalid University, 61413 Abha, Saudi Arabia; 3 Department of Chemistry, Chandigarh Engineering College, Chandigarh Group of Colleges-Jhanjeri, Mohali, Punjab 140307 India; 4 School of Chemical Engineering, Yeungnam University, Gyeongsang, 38541 Republic of Korea; 5 Department of Research and Innovation, Saveetha School of Engineering, SIMATS, Chennai, Tamil Nadu 602105 India; 6 Department of Peace and Development Studies, Njala University, Bo Campus –18, Freetown, Sierra Leone; 7 University Center for Research & Development (UCRD), Chandigarh University, Mohali, Punjab, India

**Keywords:** Entropy generation, Nusselt number, Numerical simulation, Thermal conductivity, Thermohydraulic performance, Viscosity, Engineering, Materials science, Nanoscience and technology

## Abstract

This study investigates the heat transfer performance of water-based Al_2_O_3_-TiO_2_ (50:50) hybrid nanofluids under turbulent flow conditions. Al_2_O_3_ and TiO_2_ nanoparticles (13 and 21 nm, respectively) were dispersed in water to prepare the nanofluids in the concentrations range of 0 to 1 vol%. Thermal conductivity and viscosity are measured in the temperature range of 30 to 60^o^C for the prepared nanofluids. Experimental and numerical analyses explored the effect of concentration and Reynolds number on Nusselt number, entropy generation, and friction factor. The results demonstrate that maximum viscosity enhancement of 15.77 and 14.76% is observed for 1 vol% of hybrid nanofluid and Al_2_O_3_ nanofluid compared to base fluid at 30 ^o^C, respectively. The maximum Nusselt number enchantment is 70.4% for 1 vol% of hybrid nanofluid compared to the water. Similarly, hybrid nanofluids achieved a remarkable reduction in total entropy generation of 46% in contrast to the base fluid. New correlations are proposed to predict both the Nusselt number and friction factor for hybrid nanofluids. Furthermore, employing machine learning techniques, highly accurate models are developed. These findings highlight the promising role of hybrid nanofluids in achieving efficient thermal management in various applications.

## Introduction

The expansion of industrial activities in recent decades has created a substantial demand for energy resources. With advanced research, heat transfer technologies are continuously getting better^1^. Although considerable attempts are made to improve heat transfer, the performance is still constrained by the poor TC of common heat transfer fluids (HTFs), like ethylene glycol, water, and oil^2^. A new class of HTFs known as nanofluids (NFs) was developed and introduced by Choi^3^ as a result of the improvement in material engineering, which has made it possible to produce nano-sized particles (particle size less than 100 nm). Due to their superior thermo-physical characteristics, particularly in terms of TC, these fluids enhance heat transfer^4,5^. The total surface area of particles increases when metallic and non-metallic nanoparticles (NPs) are added to the base fluids, increasing the rate of heat transfer in comparison to the base fluids^6^.

Although it has been demonstrated that adding NPs accelerates the thermal boundary formation leading to improvement in the base fluid’s thermal characteristics, the heat transfer enhancement is still regarded as minimal. As a result, hybrid nanofluids (HNFs) are the subject of recent studies. These fluids can be produced by mixing two or more unique NPs into the base fluid^7^. Most studies believe that HNF has superior thermal characteristics to mono NFs. Furthermore, regarding flow behavior, the mono NF and HNF might raise the host fluid’s pressure drop $\:(\varDelta\:p)$ and friction factor. The VST increased with NF concentration, which in turn increased the $\:\varDelta\:p$^8^ and, consequently, the penalty of pumping power^7,9,10^.

Researchers are increasingly focusing on unravelling the intriguing thermal and hydrodynamic properties of ternary NFs, which combine the advantages of three distinct NPs^11–13^. Prior research has established the promise of ternary NFs. Ekiciler^14^ demonstrated that their effectiveness increases with pipe wall corrugation. Additionally, numerical studies have comprehensively analyzed various heat transfer and flow parameters, including the key performance metric, friction factor, Nusselt number (Nu), temperature distribution, and velocity patterns^15,16^. Mousavi et al.^17^ determined the heat transfer and ∆ of water-based various mono, hybrid, and ternary NFs comprising CuO/CaCO_3_/SiO_2_ through a horizontal circular pipe under a turbulent flow regime. The maximum augmentation in heat transfer coefficient (HTC) was observed for ternary fluids.

Shahsavar et al.^18^ investigated the heat transfer and entropy production features of a water-based HNF incorporating Fe_3_O_4_ and CNT NPs in their numerical analysis. The research concentrated on natural convection flow inside a concentric annulus. The researchers discovered that increased NP concentrations resulted in greater rates of frictional and thermal entropy formation at varying Rayleigh numbers. Boruah et al.^19^ calculated the heat and entropy production in a micro-channel for 2-D, laminar, incompressible, single-phase flow of Al_2_O_3_ NF. They discovered that the average Nu rises with Reynolds number (Re) for all NF concentrations. Seyyedi et al.^20^ explored the heat transfer and entropy generation of a hexagonal cavity with Copper NF owing to the orientated magnetic field. According to the findings, aspect ratio enhances both average Nu and entropy generation.

Chinakwe et al.^21^ investigated the effects of angle of inclination, Re, and concentration upon the thermal along with hydraulic behavior, as well as entropy generation rates of Al_2_O_3_ NFs passing through a smooth circular aluminum pipe under turbulence conditions in their computational study. The results showed that the tube orientation at + 45^o^ had the greatest thermal variances when compared to the other orientations. From their experimental results, Sundar et al.^22^ and Kanti et al.^23^ concluded that frictional entropy generation has less effect on total entropy generation (TEG).

Several studies have explored innovative approaches to enhance fluid dynamics and heat transfer performance. Li and Li^24^ examined the behavior of non-Newtonian fluids in microchannels under the influence of electromagnetohydrodynamic forces, providing insights into flow control mechanisms at the microscale. Ma et al.^50^ developed a multi-objective topology optimization framework to improve the efficiency of cooling elements, ensuring better thermal management. Long et al.^25^ introduced a laser-based method to create hierarchical structures on copper surfaces, inspired by rose petals, to enhance bubble nucleation and detachment. Meanwhile, Wu et al.^26^ conducted experimental investigations to refine mini-groove geometries and optimize micro-nano composite porous layers, aiming to improve capillary-fed boiling heat transfer.

Traditional approaches for examining the thermophysical properties based on first principles frequently depend on statistical mechanics, kinetic theory, or effective medium theory (EMT). Although these methodologies are based on robust theoretical frameworks, they may be very complex, computationally taxing, and need substantial experimental validation, which is both time-consuming and expensive. Recently, machine learning (ML) has become a feasible and effective method for accurately modeling and simulating thermophysical characteristics^27,28^. The increasing incorporation of machine learning in scientific and technical fields indicates a significant transformation, providing novel opportunities for predictive analysis. Diverse machine learning algorithms, including neural networks, random forests, and support vector machines, have exhibited exceptional versatility in identifying intricate patterns within data^29^. Researchers currently extensively utilize these soft computing methodologies to forecast experimental thermophysical parameters and heat transport characteristics with improved efficiency. Utilizing machine learning techniques may markedly enhance predicted accuracy while minimizing computing effort and time, rendering them an indispensable asset for progressing study in this field.

The development of efficient HTFs is crucial for advancements in various fields, including thermal management of electronics, energy conversion systems, and transportation. While research has explored HNFs, knowledge gap remains regarding the thermal performance and entropy generation of Al_2_O_3_-TiO_2_ HNFs. Our work aims to bridge this gap and contribute to the development of next-generation HTFs.

The study employs a comprehensive approach, integrating numerical simulations, experimental analysis, and machine learning models to evaluate the effect of hybrid nanofluid concentration and Re on Nu, entropy generation, and friction factor. The study investigates the flow behavior as well as convective heat transfer for water-based Al_2_O_3_-TiO_2_ (50:50) HNFs inside a circular tube subjected to a constant heat flux of 8625 Wm^− 2^. The HNFs were prepared with concentrations varying from 0 to 1 vol%. Initially, numerical simulations were conducted using ANSYS Fluent, solving the Navier-Stokes and energy equations with a k-ε turbulence model. Mesh independence tests and validation with standard fluids ensured accuracy. The experimental setup, consisting of a calibrated flow system with temperature and pressure sensors, was then used to validate the numerical results. Hybrid nanofluids were prepared using Al₂O₃-TiO₂ with Sodium Dodecylbenzene Sulfonate (SDBS) as a surfactant, and their stability was confirmed through zeta potential analysis. A unique 5/2 Matern Kernel-based Gaussian Process Regression (GPR) technique was employed to develop prognostic models for entropy generation and heat transfer characteristics based on the experimental data.

## Materials and methods

### Nanofluid preparation

The required NPs, both sourced from Sigma-Aldrich, were acquired through Synergy Scientific Sdn Bhd. The Al₂O₃ particles had an average size of less than 13 nm, while the TiO₂ particles averaged 25 nm in diameter^24^. Please refer to our article^30^ for the characterization of NPs. A PGB-3010 model and Wensar make digital balance were employed to measure a desired quantity of nanoparticles estimated using Eqs. (1) and (2)^30^. The NFs are synthesized by a two-step method. Initially, spherical Al_2_O_3_, Al_2_O_3,_ and TiO_2_ NPs were dispersed in 60 mL base fluid (water) in a ratio of 50:50 to prepare different concentrations of NF.

SDBS was chosen as the surfactant for its strong dispersing ability, preventing NP agglomeration and enhancing the stability of HNFs. Its anionic nature improves electrostatic repulsion between HNFs, ensuring uniform dispersion, and making it widely used in nanofluid preparation. We incorporated SDBS in varying concentrations from 0 to 0.2 of the NP weight for mono NF whereas for HNF it is found to 0 to 0.3 times of the total hybrid NPs weight, increasing in 0.05 times. At 0.2 and 0.3 times the NPs weight, both mono and HNFs exhibited the highest zeta potential, indicating optimal stability. Based on this, we maintained the same proportion for our study. After adding the NPs to the water, SDBS was introduced and agitated. Subsequently, it was stirred for one hour in a magnetic-type stirrer (REMI 2MLH) at the speed of 1000 pm. It was then maintained for four hours in ultrasonication (400 W, 24 kHz) to break down particle aggregation and obtain long-term dispersion^30^.

1 $$\:\:\:\text{F}\text{o}\text{r}\:\text{A}{\text{l}}_{2}{\text{O}}_{3}\:\text{N}\text{F},\:\:\text{V}\text{o}\text{l}\text{u}\text{m}\text{e}\:\text{c}\text{o}\text{n}\text{c}\text{e}\text{n}\text{t}\text{r}\text{a}\text{t}\text{i}\text{o}\text{n}\:\left(\phi\:\right)=\:\frac{\left(\frac{{\text{m}}_{np1}}{{{\uprho\:}}_{np1}}\right)}{\left(\frac{{\text{m}}_{np1}}{{{\uprho\:}}_{np1}}\right)+\left(\frac{{\text{m}\:}_{\text{w}}}{{{\uprho\:}\:}_{\text{w}}}\right)}$$

2 $$\:\:\text{F}\text{o}\text{r}\:\text{H}\text{N}\text{F},\:\:\text{V}\text{o}\text{l}\text{u}\text{m}\text{e}\:\text{c}\text{o}\text{n}\text{c}\text{e}\text{n}\text{t}\text{r}\text{a}\text{t}\text{i}\text{o}\text{n}\:\left(\phi\:\right)=\:\frac{\left(\frac{{\text{m}}_{np1}}{{{\uprho\:}}_{np1}}\right)+\left(\frac{{\text{m}}_{np2}}{{{\uprho\:}}_{np2}}\right)}{\left(\frac{{\text{m}}_{np1}}{{{\uprho\:}}_{np1}}\right)+\left(\frac{{\text{m}}_{np2}}{{{\uprho\:}}_{np2}}\right)+\left(\frac{{\text{m}}_{\:\text{w}}}{{{\uprho\:}}_{\:\text{w}}}\right)}$$

Where np1 and np2 are the Al_2_O_3_ and TiO_2_ NPs, correspondingly. m, $\:{\uprho\:}$ and w denote mass, density and water, individually.

### Thermophysical properties

Table 1 presents the thermophysical properties of NPs and NFs used in this work. Please refer to our recently published article for information on the thermophysical characteristics of test NFs^30^. Based on the mixture law relations shown in Eqs. (3) and (4), we have determined the density and specific heat of NFs^31,32^.

3 $$\:{{\uprho\:}\:}_{\text{h}\text{n}\text{f}}={{\upphi\:}}_{\text{n}\text{p}1\:}{{\uprho\:}}_{\text{n}\text{p}1}+\:{{\upphi\:}}_{\text{n}\text{p}2\:}{{\uprho\:}}_{\text{n}\text{p}2}+(1-{{\upphi\:}}_{\text{h}\text{n}\text{f}}){{\uprho\:}}_{\text{w}}$$

4 $$\:{\text{c}}_{\text{p},\:\:\text{h}\text{n}\text{f}}\:=\:\:\frac{({\uprho\:}{{\upphi\:}\text{c}}_{\text{p}}{)}_{\text{n}\text{p}1}+\:({\uprho\:}{{\upphi\:}\text{c}}_{\text{p}}{)}_{\text{n}\text{p}2}+\:\left({1-{\upphi\:}}_{\text{h}\text{n}\text{f}}\:\right)({\uprho\:}{{\upphi\:}\text{c}}_{\text{p}}{)}_{\text{w}}}{{{\uprho\:}}_{\text{h}\text{n}\text{f}}}\:\:$$

**Table 1:** Thermophysical properties of NPs at 30 ^o^C.

Specifications	TiO_2_^30,33,34^	Al_2_O_3_^23,30,33,35^
Purity	99.5	99.8
Particle size (nm)	21	13
⍴, kg m^− 3^	4250	3960
$\:{\text{C}}_{\text{p}}$, J/ kg.K	686.2	773
K, W/m. K	8.9	36

Where

$$\:{{\upphi\:}}_{\text{h}\text{n}\text{f}}=\:{{\upphi\:}}_{\text{n}\text{p}1\:}+\:{{\upphi\:}}_{\text{n}\text{p}2\:}$$

C_p hnf_ and $\:{{\uprho\:}}_{\text{h}\text{n}\text{f}}$ specific heat and effective density of HNF, respectively.

The TC in the case of Al_2_O_3_-TiO_2_ HNFs was assessed employing a KD2 Pro thermal analyzer from Decagon Devices Inc. (USA), a widely adopted tool in NF research. Calibration was done with glycerol before measurements, which were taken over a temperature range of 30–60 °C, with five readings averaged after 20-minute intervals. Dynamic viscosity was measured using a LVDV-II Pro Brookfield Programmable viscometer within the same temperature range, with measurements repeated every 15 min for consistency. The average of the measurements is considered for the analysis.

### Test facility

**Fig. 1:**
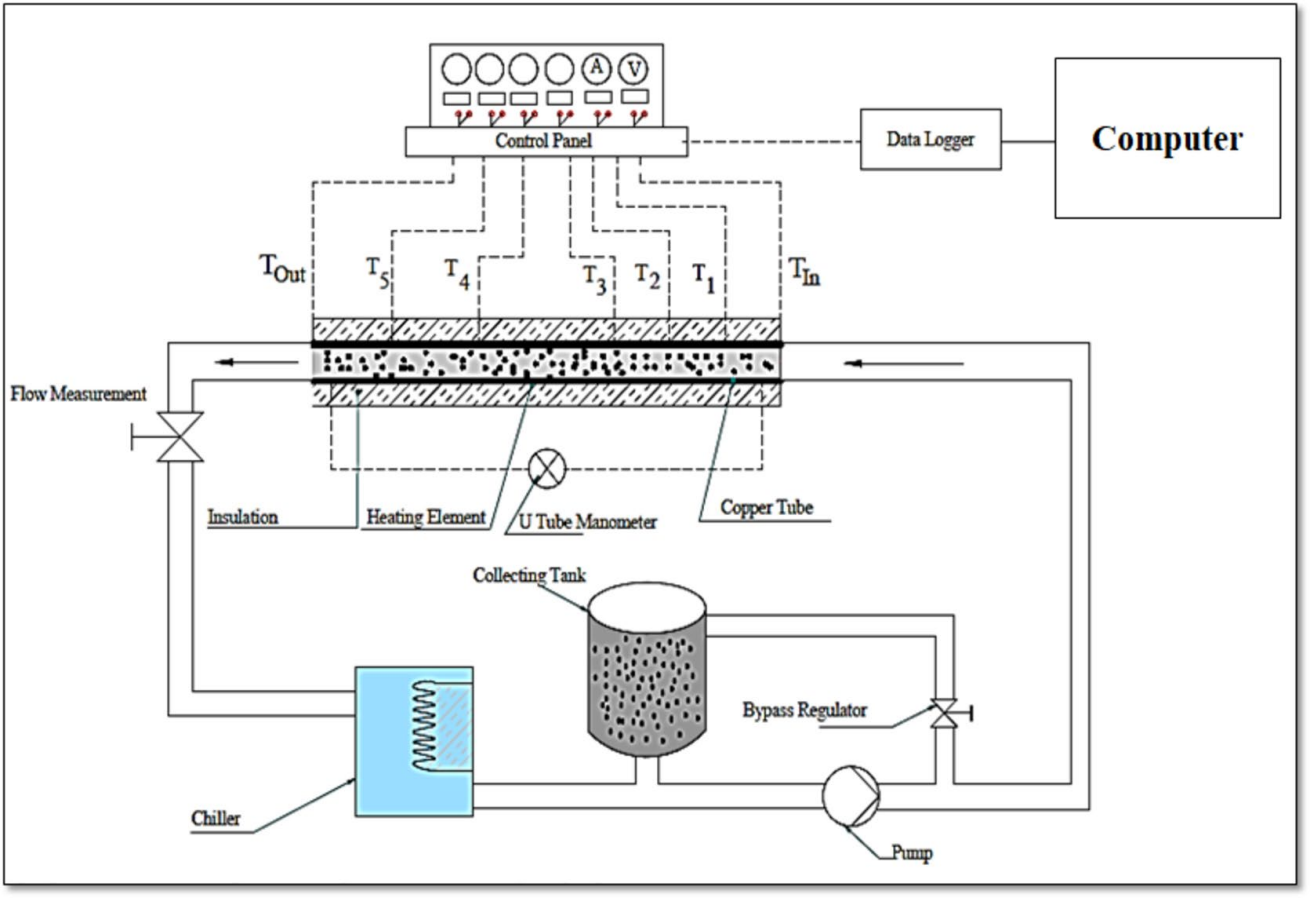
Experimental setup diagram.

Figure 1 illustrates the line diagram of the experimental test setup, which includes a test section (copper tube), pump, heating element, chiller, collector tank, and manometer (u-tube type). The test tube was 16 mm dia and 1.5 mm wall thickness with 1500 mm length^23^. To measure the temperatures, precise thermocouples (K-type) having a ± 0.1 °C uncertainty were placed suitably at the entrance of the tube entrance, then at 250 mm length followed by 250 mm gaps continuously over the entire length from the fluid inlet side^31^. The thermocouples were brazed onto the tube at the above-mentioned positions. A nichrome wire heater (Make: Omega, USA) of 1500 W capacity is used to heat the test section. The Countronics model data logger was connected with the thermocouples.

The test tube was then covered in a heating element, which was then connected to the panel. In the control panel, the variation in voltage and current is monitored. Insulation made of asbestos rope covers the entire test tube. The working fluid was drawn into a 0.02 m^3^ SS collector tank using a 0.5 hp pump. The 30 °C bulk fluid temperature was attained with the help of a commercial 2000 W water chiller Voltas make “Tushar” model at any flow rate with a ± 1^o^C variation^23^. The pressure of the fluid has been recorded with a mercury-filled U-tube manometer given that it is crucial for calculating the thermohydraulic performance of a system^32^.

### Data analysis

The heat transfer coefficient (h) and Nu in the case of fluid flows were estimated with Eqs. (5) and (6), correspondingly, employing experimental data^23,31,32^:

5 $$\:\text{h}\:=\:\frac{\text{Q}}{{\text{A}}_{\text{s}}({\text{T}}_{\text{s}\:}-{\text{T}}_{\text{b}})}$$,

Where, Q = V$\:\times\:$I (heat supplied) in Watts, (5a)

5b $$\:{\text{T}}_{\text{b}}=\frac{{\text{T}}_{\text{i}}+{\text{T}}_{\text{o}}}{2}$$,

5c $$\:\:\:{\text{T}}_{\text{s}}=\frac{{\text{T}}_{1}+{\text{T}}_{2}+{\text{T}}_{3}+{\text{T}}_{4}+{\text{T}}_{5}}{5}$$

6 $$\:\text{N}\text{u}\:=\:\frac{\text{h}\:\text{D}}{\text{k}}$$

For fluids moving in a tube, it is vital to deliver dependable references to provision the outcomes of friction factor (f) and Nu of test fluids considered in this study. The Nu of water obtained from testing as well as computational fluid dynamics (CFD) -derived values are compared employing the Dittus–Boelter^36^ expression as shown in Eq. (7)^23^.

7 $${\text{Nu}}\,=\,0.0{\text{23}}.{\text{R}}{{\text{e}}^{0.{\text{8}}}}.{\text{P}}{{\text{r}}^{0.{\text{4}}}}$$

The pressure drop values are employed to estimate the Darcy friction factor employing Eq. (8). It was then comparatively analyzed with equations such as Mutalikdesai^37,38^ expression shown as Eqs. (9) and (10), respectively^32^.

8 $$\:\text{f}\:=\:\frac{\varDelta\:p}{\left(\frac{L}{D}\right)\:\left(\frac{\rho\:{V}^{2}}{2}\right)}$$

9 $$\:{\text{f}=\:(0.79\:{\text{l}\text{o}\text{g}}_{n}\:\text{R}\text{e}\:\--\:1.64)}^{-2}\:\text{s}\text{u}\text{b}\text{j}\text{e}\text{c}\text{t}\text{e}\text{d}\:\text{t}\text{o}\:3000\hspace{0.17em}<\hspace{0.17em}\text{R}\text{e}\hspace{0.17em}<\hspace{0.17em}5\times\:\:{10}^{6}$$

10 $$\:\text{f}=\frac{0.316}{{\text{R}\text{e}}^{0.25}}$$

Equation (11) denotes the TEG (S_gen, T_) of HNF in the test section as the sum of heat transfer (S_h, t_) and frictional entropy (S_f, t_) generation^22,31,32^.

11 $${{\text{S}}_{{\text{gen}},{\text{ T}}}}={\text{ }}{{\text{S}}_{{\text{h}},{\text{ t}}}}+{\text{ }}{{\text{S}}_{{\text{f}},{\text{ t}}}}$$

TEG in a tube can be estimated by employing the Eq. (12)^22^.

12 $$\:{\text{S}}_{\text{g}\text{e}\text{n},\:\:\text{T}}=\frac{{\text{Q}}^{2}}{{\uppi\:}\:\text{k}\:\text{L}\:{\text{N}\text{u}\:\text{T}}_{\text{i}\text{n}}\:{\text{T}}_{\text{o}\text{u}\text{t}\:\:}}\:+\:\frac{8\:{\text{L}\:\text{m}}^{3}\text{f}\:}{{\:{\uppi\:}}^{2}\:{{\uprho\:}}^{2}{\text{D}}^{5}\left({\text{T}}_{\text{o}\text{u}\text{t}\:-}{\text{T}}_{\text{i}\text{n}\:}\right)}\:\text{l}\text{n}\:\left(\frac{{\text{T}}_{\text{o}\text{u}\text{t}\:\:}}{{\text{T}}_{\text{i}\text{n}\:\:}}\right)$$

The Bejan number (*Be*), can be used to depict the effect of S_h, t_ as well as friction S_f, t_ over the TEG^32^.

$$\:\text{B}\text{e}=\frac{{S}_{h,\:t}}{{\text{S}}_{\text{g}\text{e}\text{n},\:\:\text{T}}\:}$$

### Numerical model

**Fig. 2:**
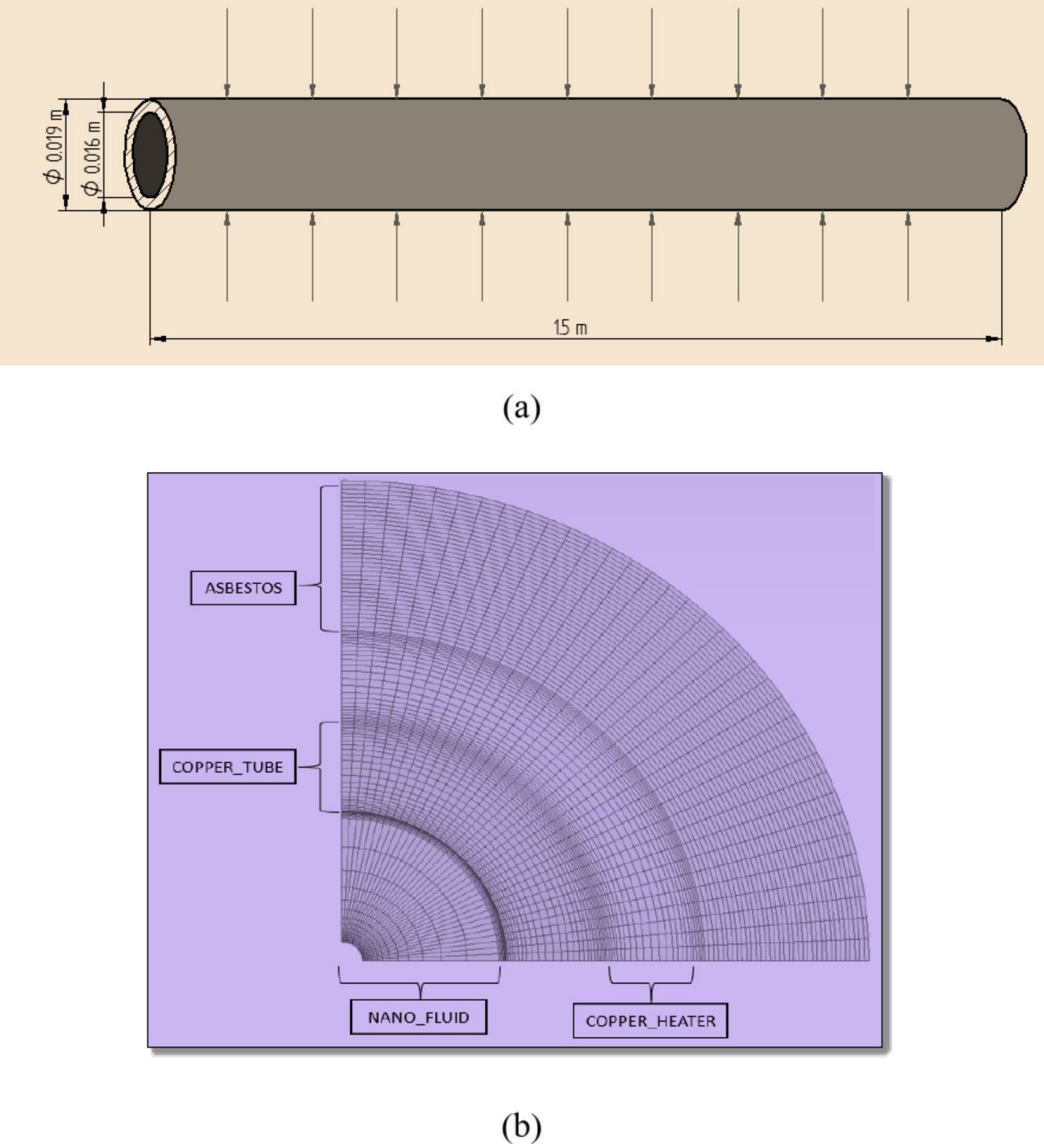
(**a**) Layout of the tube subjected to constant heat flux. (**b**) Copper tube model showing mesh.

The experimental setup comprises a dedicated domain that separates the model. The exit side of the tube was subject to exit pressure, while the inlet side had an incoming velocity based on various velocities^23^. The tube’s inlet fluid temperature was 30 °C and the test section outer wall was subject to a critical heat flux being 8625 Wm^− 2^. Using a no-slip condition, to obtain a fully developed flow, the outlet and inlet of the section under observation were expanded to five times the tube’s inner diameter^23,31^. The heat flow, which was simulated as the adiabatic type of wall, was set to 0 over the extended walls^31^. The outline of the test setup employed for modeling as well as the mesh framework, are shown in Fig. 2(a) and (b), correspondingly. The tube shape was meshed three times to confirm simulation results using water. A test mesh was employed for the CFD framework. In this work, the realizable k-ε framework using improved treatment of wall and full Y^+^ treatment was adopted^26^. The Y^+^ values in this work are < 1^31,39^. The ANSYS Fluent program was used to solve the turbulent type forced heat transfer equation using the finite volume approach. For the domains of liquids and solids, meshes of hexahedral shape boundaries were utilized^23,32^. Turbulent intensity (I), as described in Eq. (14), was utilized to estimate turbulent measures^32^.

13 $$\:I=0.16\text{R}{\text{e}}^{-1/8}$$

These assumptions were considered for the numerical study: The NF was an incompressible, steady Newtonian fluid that was not affected by gravity; NFs are thought to have constant thermophysical properties^32,40,41^. Please refer our published article for the governing equations for this numerical work^39^.

### Gaussian process regression

GPR is a probabilistic non-parametric regression technique that models the distribution over functions using a Gaussian Process. GPR is a flexible technique for regression that is especially beneficial when the underlying relationship between inputs and outputs is complicated, and there is uncertainty in the data or noise in the measurements^42^. GPR’s central premise is that the projected output value at any input location is a linear combination of the observed target value at all other input sites, weighted by a kernel function that evaluates the resemblance between the control factors^43,44^. The kernel function defines the covariance structure of the underlying Gaussian process, which determines the smoothness of the forecasted function and the predictability. To execute GPR, we must first define a kernel function that captures input similarity. The radial basis function (RBF) kernel is the most often used kernel function, and it measures the similarity between inputs based on their Euclidean distance. Additional kernel functions that can be employed include the Matern kernel and the linear kernel^44,45^.

The kernel function hyperparameters are then estimated using maximum likelihood estimation or cross-validation. These hyperparameters determine the smoothness of the predicted function and the degree of noise in the data. Lastly, we apply the trained GPR model to make predictions on additional input points and evaluate the prediction uncertainty. GPR can also be used for multi-output regression, classification, and unsupervised learning. The Gaussian Process and the kernel function used to characterize the process covariance structure are the essential mathematical elements in GPR^46,47^. The present study utilizes a Gaussian Process to describe the distribution across functions f(x) in the context of GPR, where x denotes the input variable and f(x) represents the output variable. We suppose that function values are all jointly Gaussian^42,48,49^.

14 $$\:f\left(x\right)\sim\:GP\left(p\left(x\right),\:q\left(x,\:{x}^{{\prime\:}}\right)\right)\:.$$

Herein, the p(x) denotes the mean function while the covariance function is shown with $\:q(x,\:x{\prime\:})$ as the covariance function.

The mean function indicates our prior knowledge about the function type, whereas the covariance function specifies a function’s smoothness and the degree of correlation amongst the function’s various values input points. the kernel function $\:q(x,\:x{\prime\:})$ must now be specified. The kernel function computes the covariance matrix $\:Q$, where $\:Qij\:=\:q$, by measuring the similarity between input points (xi, xj). GPR can employ a variety of kernel functions, together with linear kernel, RBF kernel, and the Matern kernel.

The 5/2 Matern kernel is a kernel function frequently employed in GPR to describe functions with continuous second derivatives. The signal variance $\:\left({\sigma\:}^{2}\right)$ and the length scale parameter$\:\:l$, which regulates, the smoothness of function, are two of the hyperparameters of 5/2 Matern. The kernel grows smoother as the length scale increases and the correlation between function values at different input locations drops. Since it strikes an appropriate mix between flexibility and computational tractability, the 5/2 Matern kernel is a common choice in GPR. It enables modeling a wide range of functions, including those with sharp changes or kinks, while also being computationally efficient to evaluate and invert. The mean function indicates our prior knowledge about the function’s form, whereas the covariance function specifies the function’s smoothness and the degree of correlation amongst the function’s various values input points^50,51^.

## Results and discussion

### Thermophysical properties

**Fig. 3:**
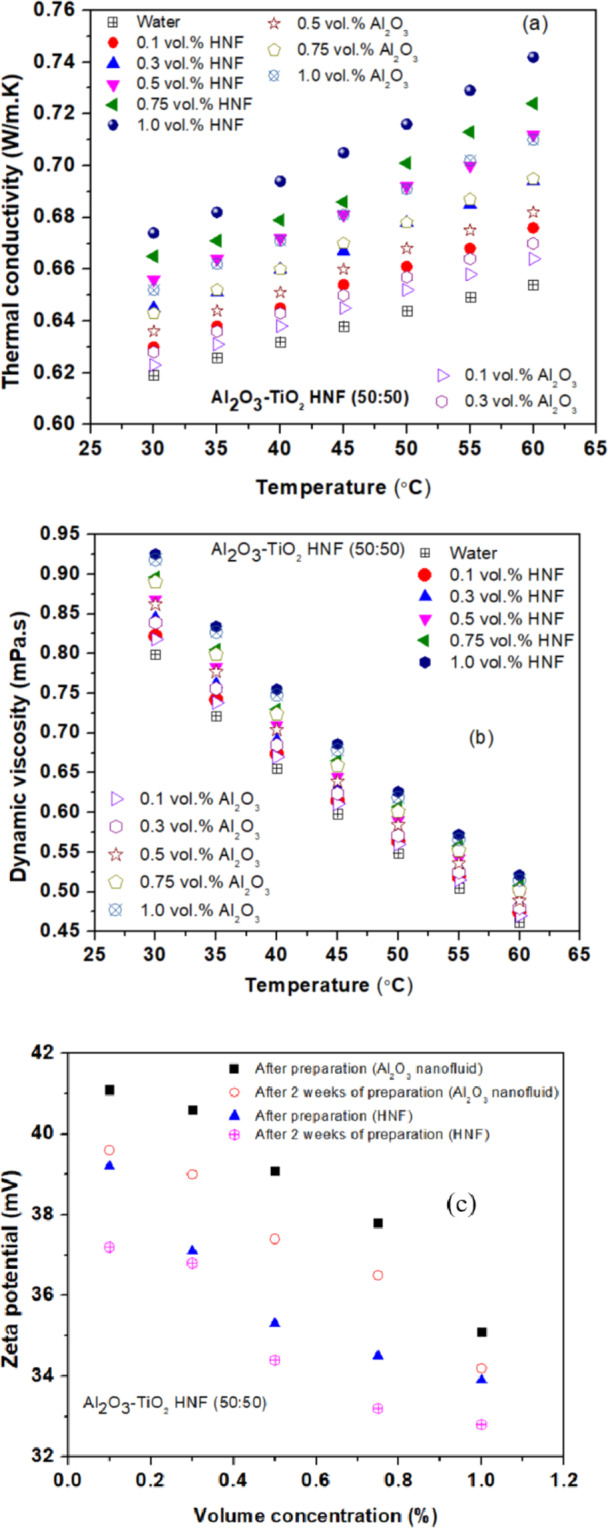
Variation of (**a**) TC and (**b**) VST with temperature. (**c**) Changes in Zeta potential for nanofluids in response to volume concentration.

Figure 3 (a) presents the variation of TC of both NFs with temperature and concentration. It is noticed that the TC of both NFs rises with temperature and concentration. As the temperature increases, the VST of the base fluid decreases, which enhances the mobility of NPs and allows for better heat transfer. The additional thermal energy at higher temperatures boosts the vibrational motion of NPs, improving phonon transport and thus TC. Furthermore, reduced VST at elevated temperatures allows for a more uniform dispersion of NPs, leading to more efficient heat conduction. Increasing the NPs concentration also contributes to higher TC, as more particles provide additional pathways for heat transfer. Moreover, at higher concentrations, the interactions between NPs become more significant, leading to improved TC through better alignment and distribution of particles in the fluid.

The enhanced TC of the Al₂O₃ (13 nm)-TiO₂ (21 nm) HNF compared to the Al₂O₃ NF alone can be attributed to several factors. The blend of Al₂O₃ and TiO₂ NPs creates a synergistic effect, where the distinct thermal characteristics of each type of NP work together to improve heat transfer. The HNF benefits from an increased surface area for heat conduction due to the smaller Al₂O₃ particles, while the slightly larger TiO₂ particles help fill thermal gaps. Additionally, the combination promotes better dispersion and stability of the NPs in the fluid, minimizing agglomeration and achieving a more even distribution. Improved phonon transport and reduced phonon scattering, facilitated by the interaction of the two types of NPs, further enhance the TC of the HNF.

Figure 3 (b) presents the variation of VST of both NFs with temperature and concentration. It is noticed that the viscosity of both NFs rises with increased particle concentration and falls with higher temperatures due to several reasons. As the concentration of NPs grows, the NF’s VST increases because more NPs lead to greater interactions between particles and between particles and the fluid, causing higher internal friction and resistance to flow. Conversely, as the temperature increases, the VST of the base fluid decreases because the thermal energy reduces intermolecular forces, allowing the fluid to flow more easily. Thus, higher NP concentrations result in higher VST, while elevated temperatures reduce VST.

The VST of the Al₂O₃ (13 nm)-TiO₂ (21 nm) HNF is higher than that of the Al₂O₃ NF due to several contributing factors. The inclusion of TiO₂ adds complexity to the NF’s particle interactions, as the combination of different NP types increases the frequency of interactions between particles and with the fluid. This results in heightened internal friction and a more significant resistance to flow. Additionally, the different sizes of the Al₂O₃ and TiO₂ particles lead to a more intricate network within the fluid, further disrupting its structure and increasing VST compared to a nanofluid composed solely of Al₂O₃ particles.

A higher (> 30 mV) zeta potential usually signifies better NF stability because it reflects strong electrostatic repulsion between particles, which helps keep them dispersed and prevents aggregation. Conversely, a lower (< 30 mV) zeta potential indicates weaker repulsion and greater risk of particle clumping, leading to potential instability. Figure 3 (c) illustrates the zeta potential for both the NFs. As concentration increases, the stability of NFs often decreases due to the higher probability of particles coming into close contact and forming clusters or aggregates that cause sedimentation. Al₂O₃ NFs typically show greater stability than Al₂O₃-TiO₂ HNFs, as the latter features NPs with different sizes and surface properties, complicating their dispersion and making it harder to prevent aggregation. The zeta potential in HNFs is often lower, signifying reduced electrostatic repulsion and, consequently, lower stability. In contrast, Al₂O₃ NFs tend to have more consistent characteristics, leading to a higher zeta potential and improved stability.

### Mesh independence test

**Fig. 4:**
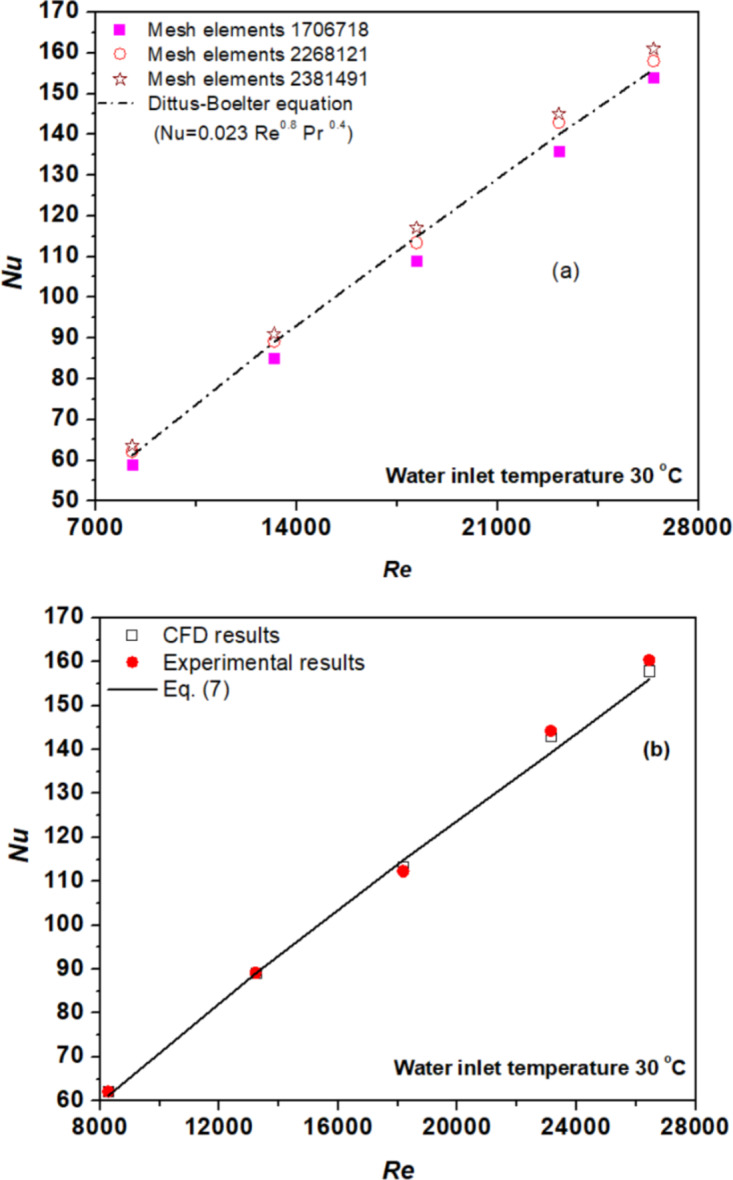
(**a**) Test of mesh independence and (**b**) Nusselt number validation for water.

The boundary layer meshes were meticulously resolved for both liquid and solid domains, ensuring accurate representation. Tube geometries underwent three iterations of hexahedral meshing, aimed at validating results against the base fluid. Comparison of CFD-derived Nu data for water with calculations from Eq. (7) across various meshes (depicted in Fig. 4(a)) revealed slight discrepancies: 5.35, 1.27, and − 3.75% for the first, second, and final meshes, respectively. Opting for computational efficiency, the second mesh was chosen for subsequent analyses. In Fig. 4(b), Nu of water estimated via CFD, and experiments were compared with those derived from Eq. (7). The maximum deviations between Eq. (7) and CFD as well as experimental data were − 2.6 and − 3.5%, respectively, indicating acceptable agreement. These findings emphasize the validity of both experimental and computational approaches, affirming the reliability of the proposed methodology.

### Heat transfer

**Fig. 5:**
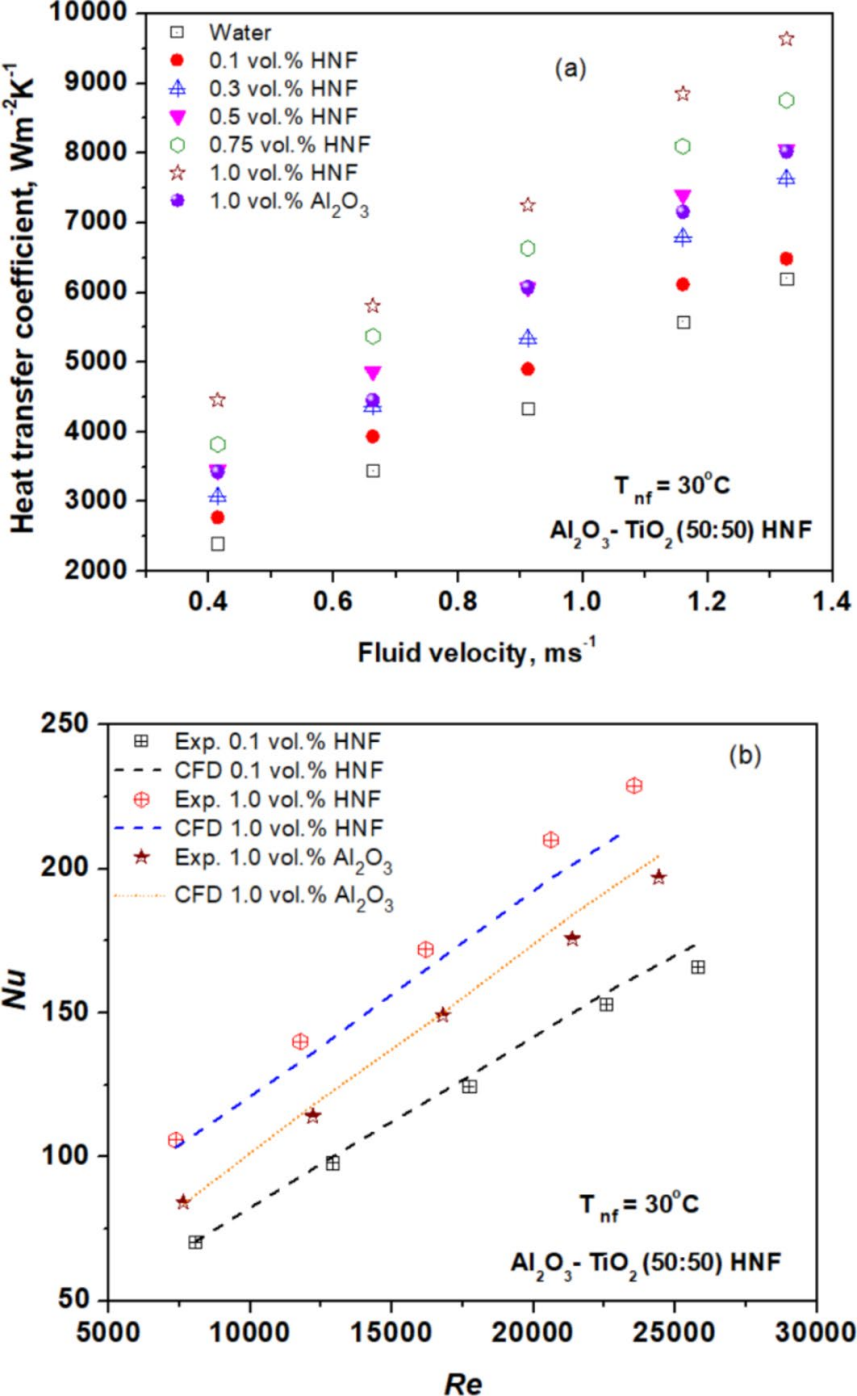
(**a**) Variation of HTC with fluid velocity of nanofluid. (**b**) Variation of hybrid nanofluid Nu with Re.

Figure 5 illustrates the influence of fluid velocity on experimental HTC outcomes. Notably, HTC with HNF surpasses that with Al_2_O_3_ NF and water at the same velocities. HNF enhances the HTC by 85.53% at 1.0 vol% and 15.43% at 0.1 vol%, compared to the base fluid, while Al_2_O_3_ NPs improve the HTC by 42.74% at 1.0 vol% as compared to the base fluid water. The improvement is attributed to the increasing TC and intensified Brownian motion induced by NPs, facilitating superior fluid mixing in both near-wall and core regions. Moreover, the utilization of TiO_2_ NPs alongside Al_2_O_3_ NPs results in increased TC due to enhanced NP mixing and delayed boundary layer development^52,53^. These factors contribute significantly to our study’s findings, elucidating the augmentation of heat transfer.

Figure 5 (b) portrays the variation in Nu with changes in Re of NFs. The results highlight that the Nu of HNF improves with concentration and Re. Moreover, at constant Re, the Nu of hybrid NF surpasses that of Al_2_O_3_ NFs. This enhancement is attributed to particle migration, which significantly influences the thermophysical properties and Brownian motion of the hybrid NPs^32^. The maximum increase in Nu reaches 25.73% compared to Al_2_O_3_ NF at 1 vol%. Additionally, Fig. 5 (b) contrasts CFD and experimental results for Nu across different concentrations at varying Re. Notably, the variance between analytical and CFD findings for Nu is negligible, as depicted in Fig. 5(b).

### Friction factor and pressure drop

**Fig. 6:**
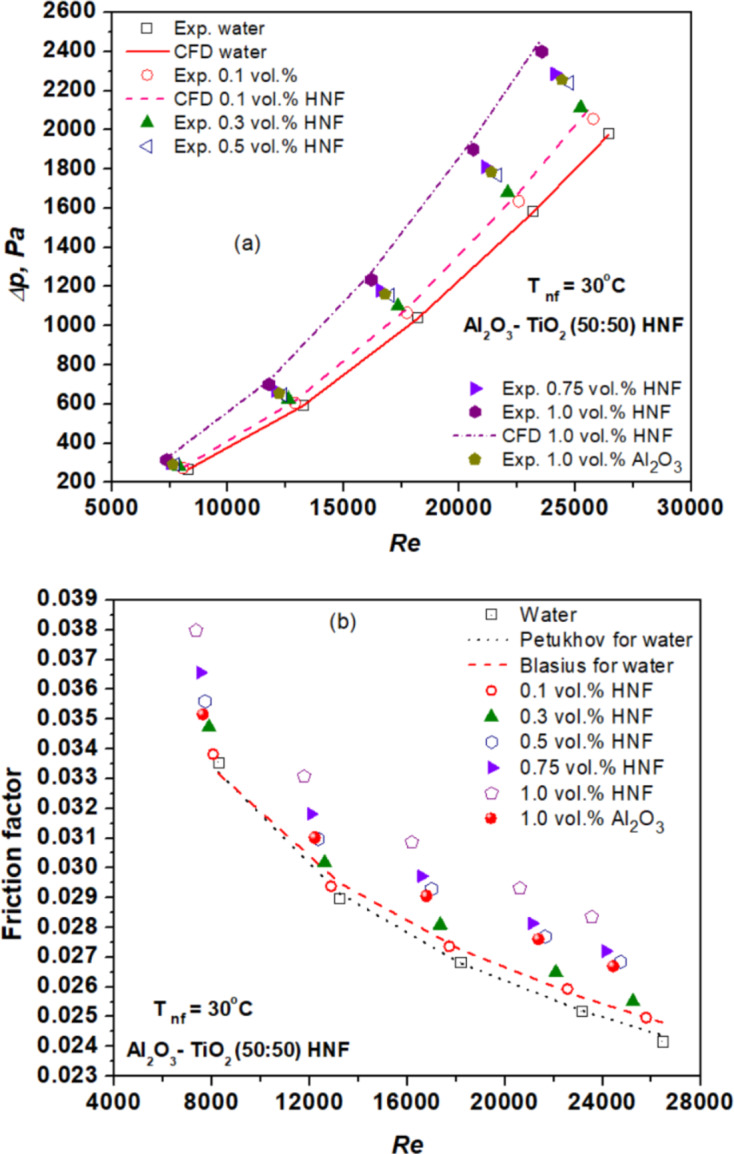
(**a**) Variation of HNF pressure drop with Re. (**b**) Variation of friction factor of HNF with Re.

Figure 6 (a) depicts the variation in *∆p* of the considered fluids in response to Re. Notably, *∆p* experiences significant amplification with increasing Re across all considered fluids. For instance, experimental *∆p* rises from 269 to 1984 Pa for water within the Re range of 8263 to 26,444. In the case of NFs, higher concentrations result in even greater *∆p* compared to water. This enhancement in *∆p* is attributed to the higher VST of nanofluids along through turbulence induced by introduction of NPs^18,22^. Furthermore, the *∆p* of HNFs exceeds that of Al_2_O_3_ NF, owing to the presence of TiO_2_ NPs which enhance the density and VST forces compared to water, especially when compared to Al_2_O_3_ NPs^30^. The maximum and minimum *∆p* augmentations in HNF of concentrations of 1.0 and 0.1 vol% are observed to be 21 and 1.12%, respectively, compared to water. Additionally, the highest and lowest *∆p* enhancements of Al_2_O_3_ NF are 13.66 and 7.81% at 1 vol% compared to water.

A notable drawback of employing NFs within tubes is the associated rise in friction factor, leading to a penalty in flow resistance. To validate the estimated friction factor using Eq. (8) for the base fluid, data from Petukhov^37^ and Blasius^38^ are utilized. Experimental friction factors from this study are compared to estimated values from Petukhov^37^ as well as Blasius^38^ equations in Fig. 6(b). The experimental friction factor exhibits a deviation of 1 and 2.64% from the estimates of Petukhov^37^ and Blasius^38^, respectively. The friction factor of NFs is estimated using Eq. (8), with outcomes depicted in Fig. 6 (b). It is observed that the friction factor increases with higher NP concentration, although decreasing with increasing Re^22,32^. The introduction of HNF leads to a rise in the friction factor due to increased VST and *∆p*. Moreover, the friction factor for HNF surpasses that of Al_2_O_3_ NF, attributed to the presence of TiO_2_ NPs in HNF, which amplify density, VST and *∆p*, resulting in a higher friction factor.

**Fig. 7:**
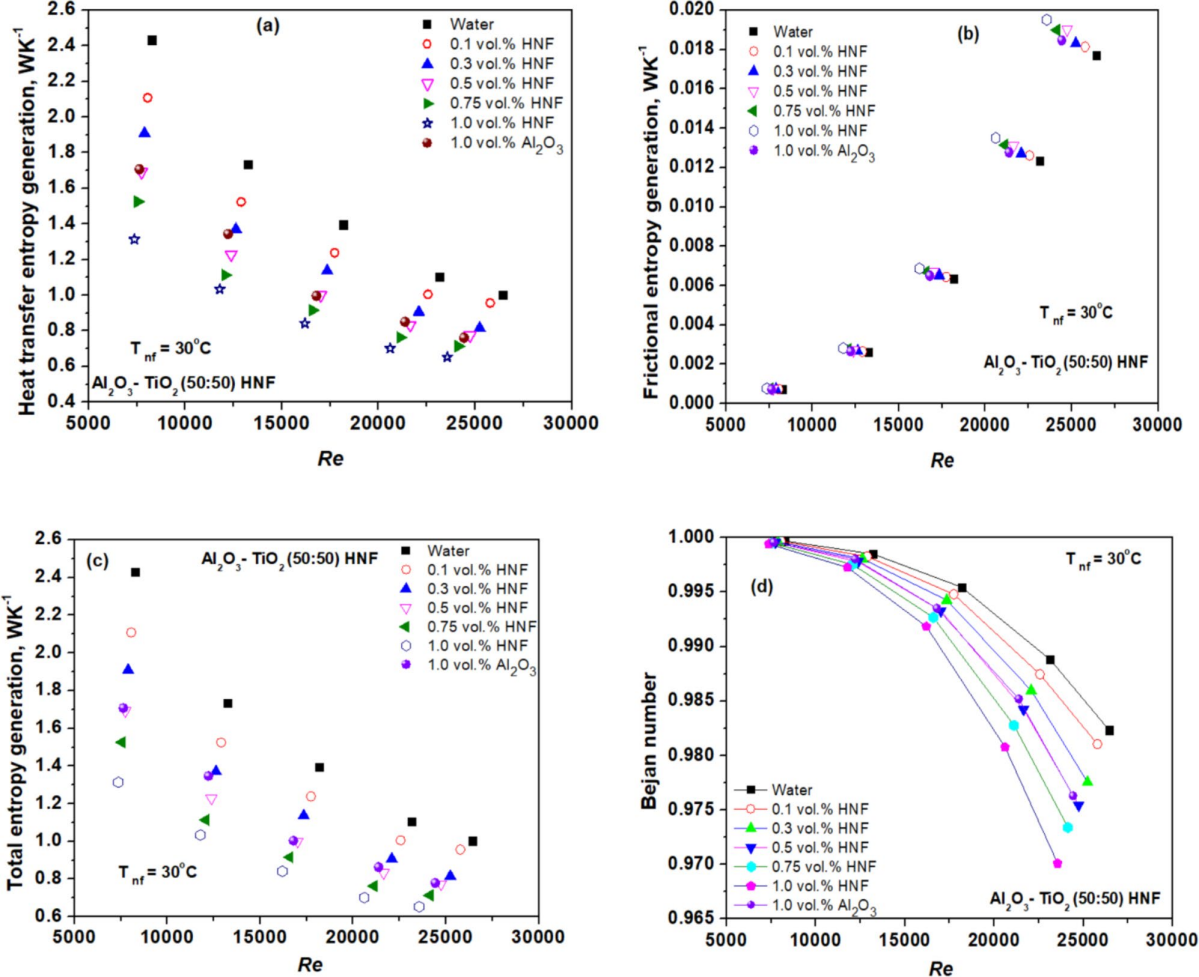
Variation of (**a**) heat transfer (**b**) frictional (**c**) total entropy generation (**d**) Bejan number of HNF with Re.

Figure 7 (a) depicts the heat transfer entropy generation of HNF at various Re. The results reveal increasing Re and concentration results in higher heat transfer entropy generation. The smallest heat transfer entropy generation occurs while the temperature variance between the fluid and the test tube wall is at its lowest level^54^. Figure 7 (a) shows that as Re and concentration increased, the heat transfer entropy generation of both water and NFs decreased^22,55^. These findings are consistent with those of Sundar et al.^22^, who revealed a decrease in heat transfer entropy generation when NFs were used instead of water. Figure 7 (b) depicts the frictional entropy generation of NF at various Re. The results demonstrate the frictional entropy generation rises with Re as well as concentration. The increase is attributed to higher levels of VST and *∆p*^32,55^.

The total entropy generation (TEG) at varying Re and concentrations is shown in Fig. 7 (c), which is the sum of heat transfer entropy generation and frictional entropy generation. The TEG decreases as Re and concentration increases^22^. The contribution of frictional entropy generation to TEG in the tube for NFs was insignificant^23,31^. As a result, the thermal effects attributed more to TEG compared with the viscous effect. Figure 7 (d) depicts the variation of Be for considered NFs at varied Re. A greater Bejan number (Be) indicates that heat transport produces additional entropy than frictional entropy generation and internal irreversibility^21,26^. Table 2 presents the uncertainty analysis of measurements carried out in this work.

**Table 2:** Uncertainty analysis of measurements

Sl. no	Property	Maximum uncertainty (%)
1	Dynamic viscosity	2.4
2.	TC	1.8
3.	Reynolds number	3.2
4.	Heat flux	0.2
5.	Nusselt number	3
6.	Friction factor	1.9

### Thermal performance factor

**Fig. 8:**
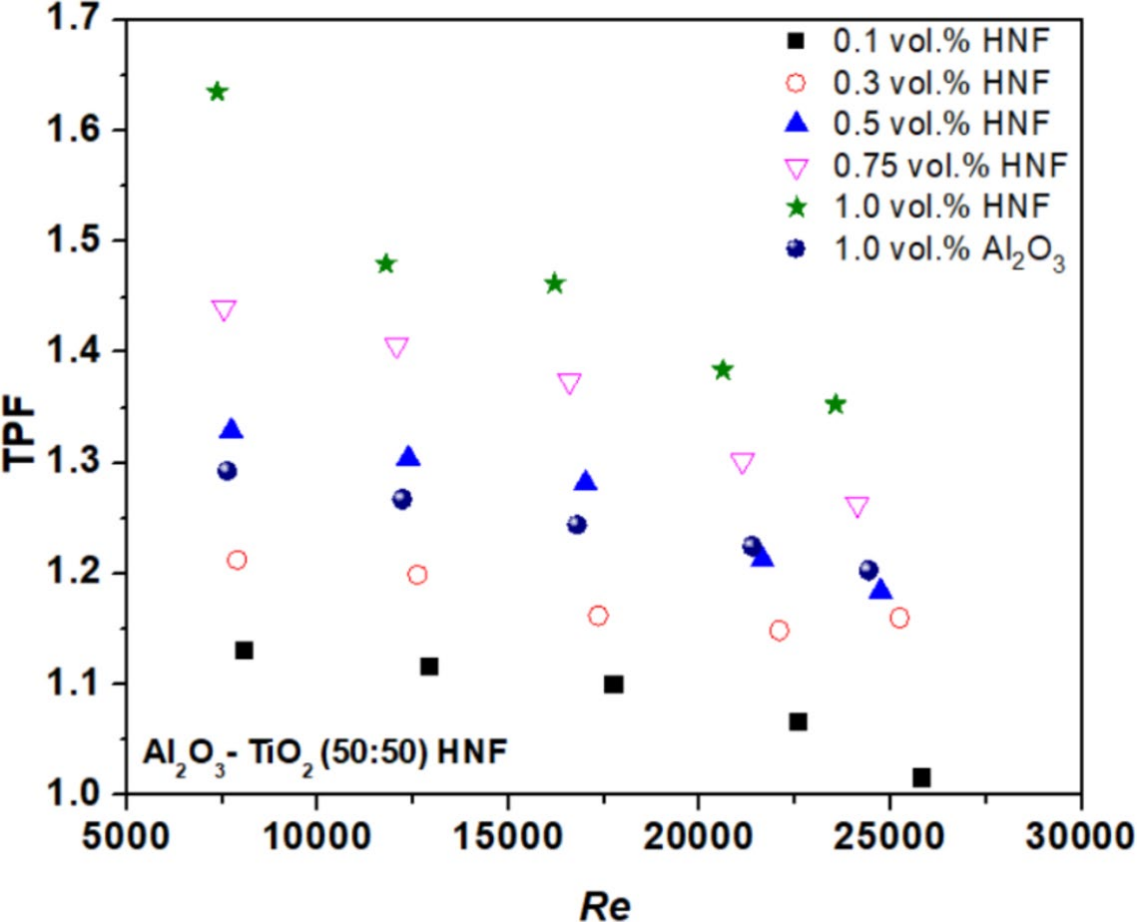
Thermal performance factor versus the Re.

The TPF of NFs is estimated using Eq. (15)^32^ and it is the ratio of amplified Nu and friction factor^31^. The values for tested NFs are depicted in Fig. 8 for the entire range of *Re*. If TPF is greater than 1, then NF may be used as the working fluid for improving the process of heat transfer. It is evident that TPF value is greater than 1 for all the developed NFs and that shows the NFs are beneficial fluids. A higher TPF was observed at higher concentrations. The TPF of HNF was found to be greater than Al_2_O_3_ NF at 1 vol%. This enhancement is because HNFs have greater TC compared to the base fluid.

15 $$\:\text{T}\text{P}\text{F}=\:\left(\frac{{\text{N}\text{u}}_{\:\text{n}\text{f}}}{{\text{N}\text{u}}_{\text{b}\text{f}}}\right)/{\:\:\:\:\left(\frac{{\text{f}}_{\text{n}\text{f}}}{{\text{f}}_{\text{b}\text{f}}}\right)}^{1/3}$$

### Model-prediction with GPR

GPR was utilized for model prediction, exploiting its adaptability in representing intricate, non-linear relationships within the dataset. Multiple open-source Python libraries, such as Pandas, NumPy, and Matplotlib, were employed for data preprocessing, visualization, and performance assessment. The Scikit-learn library enabled the execution of Gaussian Process Regression (GPR) and evaluation metrics, including MAPE, MSE, and R², to measure model precision. Correlation heatmaps were created to examine variable dependencies, ensuring a comprehensive understanding of key parameters in the prediction process.

#### Data pre-processing

The data collected during testing at various control factors have been employed for creating a prediction model with 5/2 Matern GPR. In the first stage, data pre-processing was carried out to check the health of the data. The correlation among various parameters was estimated. A data correlation matrix is a table that displays the correlation coefficients between different variables in a dataset. Correlation measures the degree and trajectory of a linear relationship between two variables. A correlation coefficient is a number between − 1 and 1, with 0 indicating no correlation, 1 indicating an ideal positive correlation, and − 1 indicating an ideal negative association. The data correlation matrix depicts the correlation coefficients between all pairs of variables in a dataset, typically in a square matrix format. The diagonal represents a variable’s connection with itself (which is always 1)^56,57^.

**Fig. 9:**
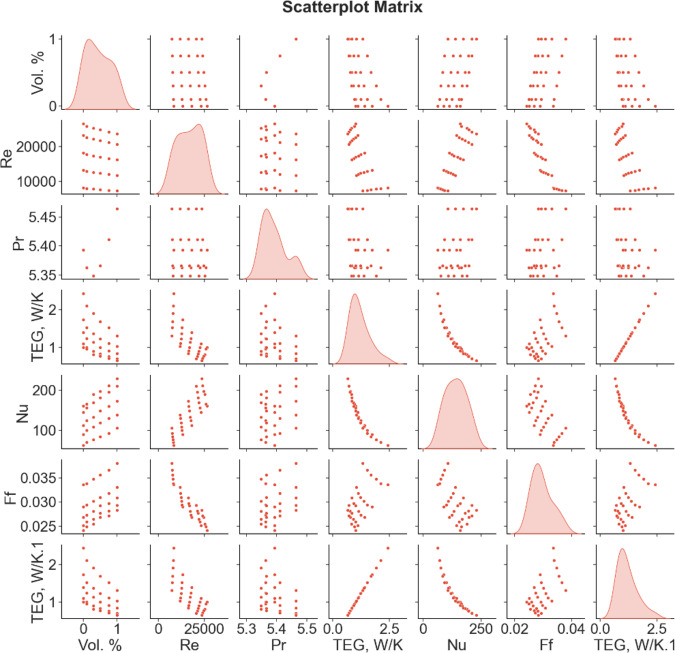
Correlation scatter plot matrix.

The correlation matrix is useful for detecting which variables are strongly related to one another and identifying links between variables. The correlation matrix for the present work is depicted in Fig. 9.

The correlation matrix depicts the correlation coefficients between five variables: NF concentration, Re, friction factor, Nu, and TEG. In the matrix, each variable is listed twice: once as the row label and once as the column label. The matrix’s diagonal line represents the correlation of each variable with itself, which is always equal to one. The correlation coefficient between each pair of variables is shown in the matrix’s off-diagonal cells. For example, the correlation coefficient for the cell at the intersection of the row for NF concentration and the column for Re is -0.136. This suggests that there is a modest negative association between these two factors.

The correlation matrix indicates that some variables have moderate correlations. For example, there is a moderately positive connection (0.451) between NF concentration and friction factor and a moderately negative correlation between Nu and TEG (-0.939). On the other hand, certain variables are weakly or not at all associated with one another. There is a weak negative association (-0.472) between NF concentration and overall entropy generation and a weak positive correlation between friction factor and Nu (0.527). It is crucial to remember that correlation does not always imply causation. Further research and testing may be required to demonstrate causal links between these variables.

#### Development of prognostic models

The friction factor, Nu, and TEG are all useful metrics for describing the behavior of NFs. The friction factor measures the barrier to the fluid’s flow in the channel or pipe, whereas ‘Nu’ measures the ‘h’ between a solid surface and fluid. TEG is a measure of the system’s overall irreversibility. Building machine learning (ML) based models for predicting these parameters in NFs entails training the algorithm with a huge experimental or simulation data dataset. The algorithm then analyses this information to find patterns and correlations between the input variables (like fluid characteristics, rate of flow, and temperature) and the desired output quantities (like friction factor, Nu, and TEG). Once trained, the algorithm can be utilized to create accurate predictions for new operating conditions or fluid properties.

**Fig. 10:**
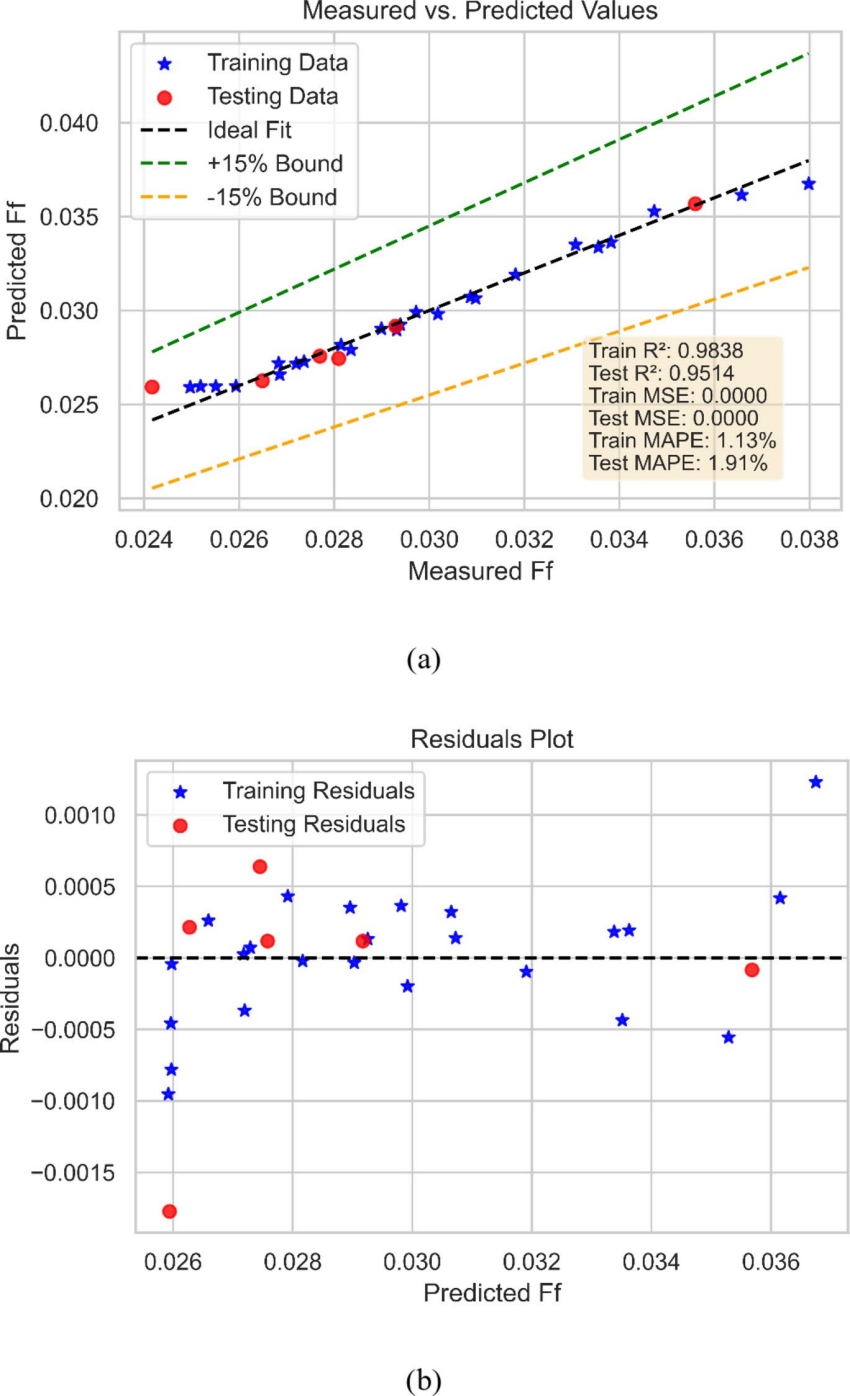
Friction factor model (**a**) Performance and (**b**) predicted v/s measured values.

The 5/2 Matern-based GPR was utilized in this study to create models for predicting three critical parameters in NF behavior: friction factor, Nu, and TEG. Figure 10 depicts the performance of the GPR model in estimating the friction factor. Figure 10(a) depicts the projected friction factor values plotted against the measured values, with the best-fit line indicated while Fig. 10(b) depicts the model’s residuals for prediction values. The results show that the 5/2 Matern-based GPR model can accurately estimate the friction factor for various operating situations and fluid parameters. The model’s overall performance is outstanding, as indicated by the close agreement between anticipated and measured friction factor values. This highlights the power of ML-based models for forecasting the behavior of complicated fluids like NFs and the significance of choosing a suitable kernel function for the GPR model.

The data supplied describes the performance metrics of the 5/2 Matern-based GPR model created for forecasting the NF friction factor. Many measures, including R^2^, MSE, and MAPE, can be employed to evaluate the model’s efficacy. R^2^ is the indicator of the correlation between expected and measured friction factor values. An R^2^ value of 0.9838 and 0.9514, respectively indicate a good positive correlation between predicted and measured values, indicating that the model can capture underlying trends in the data. MSE and MAPE are metrics of model prediction accuracy. Lower values for these indicators indicate that the model can generate more accurate predictions. The MSE value of 0 for both training and test indicates great accuracy. The MAPE of 1.13% for model training and 1.91% for model testing phase demonstrate that the model can produce accurate predictions with relatively few errors. Overall, the performance measures indicate that the 5/2 Matern-based GPR model is quite good at predicting the friction factor of NFs. The strong R and R^2^ values indicate that the model can capture the underlying trends in the data, and the low MAP and MSE values show that the model can produce accurate predictions with extremely small errors.

**Fig. 11:**
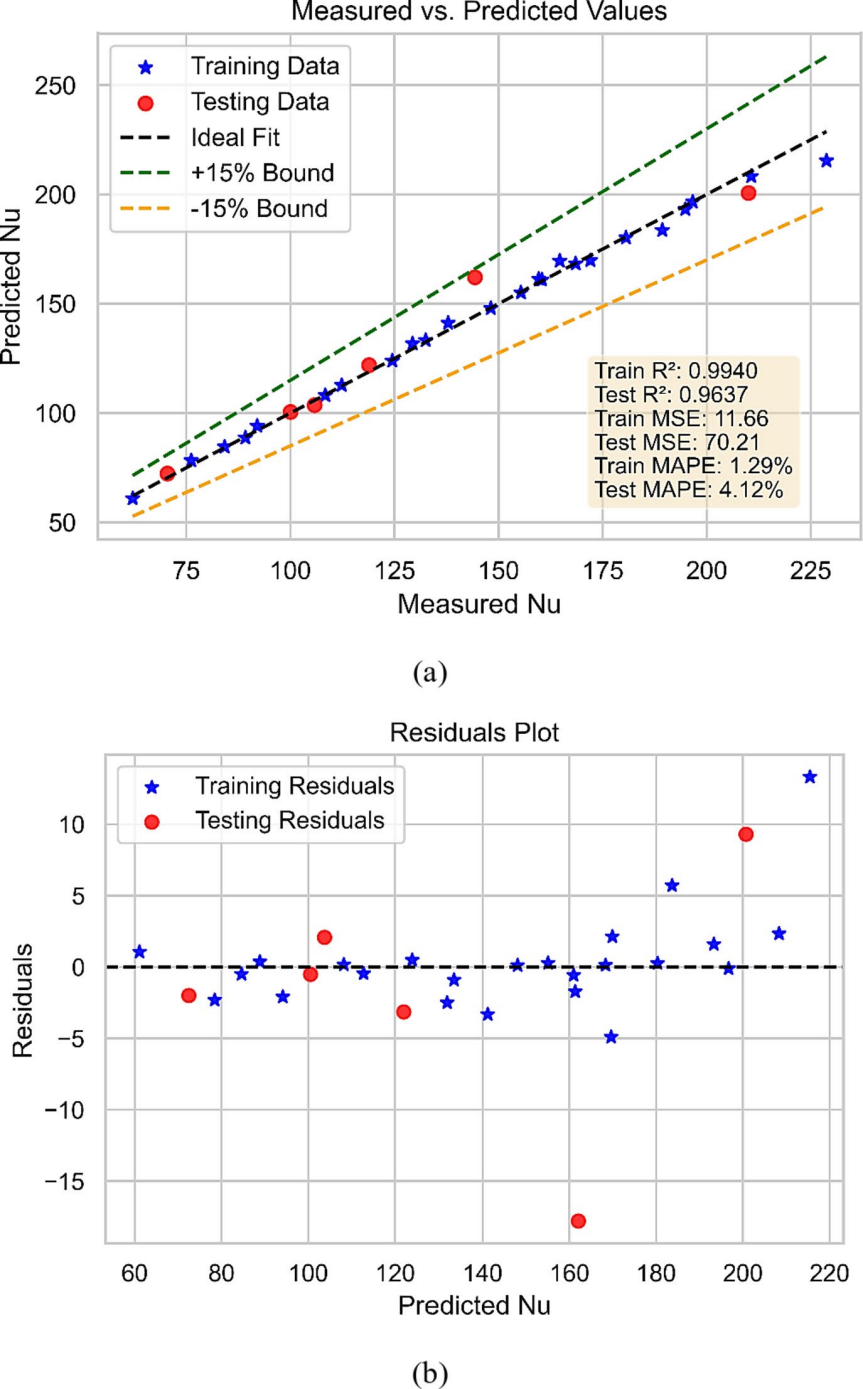
Nu model (**a**) Performance (**b**) Predicted vs. measured values.

Figure 11 demonstrates the GPR model’s performance in the prediction of Nu. Figure 11(a) depicts the projected Nu plotted against the measured values, with the best-fit line shown, whereas Fig. 11(b) illustrates residuals in model. The results demonstrate that the 5/2 Matern-based GPR model can accurately estimate the Nu for various operating conditions and fluid parameters. The model’s overall performance is excellent, as evidenced by the close agreement between predicted and measured Nu values. This demonstrates the utility of ML-based models for forecasting the behavior of complex fluids such as NFs and the importance of selecting an appropriate kernel function for the GPR model.

The performance indicators of the 5/2 Matern-based GPR model for forecasting the Nu of NFs are shown in Table 3. The evaluation metrics of the 5/2 Matérn Gaussian Process Regression (GPR) model highlight its strong predictive performance with a smooth and flexible correlation structure. Although the model shows somewhat larger error on test data, the training mean squared error (MSE) of 11.66 and testing MSE of 70.21 indicate that the model preserves a decent match on training data. Explaining almost 96% of the variation in the test dataset, the R² values of 0.994 (training) and 0.9637 (test) show that the model fairly reflects the fundamental trends. With quite small variations between predicted and actual values, the mean absolute percentage error (MAPE) of 1.29% for training and 4.12% for testing also shows great accuracy. Although the model maintains great generalizing power, the little rise in test error points to mild overfitting. The results confirm generally that the 5/2 Matérn GPR model provides consistent performance for predicting applications with smooth function approximation. These findings also suggest that ML-based models can predict the behavior of complicated fluids and provide vital insights into the fundamental physics of NFs.

**Table 3:** Statistical evaluation of developed models.

	Model training			Model testing		
Performance parameters	R^2^	MSE	MAPE	R^2^	MSE	MAPE
Friction factor	0.9838	0	1.13%	0.9514	0	1.91%
Nu	0.994	11.66	1.29%	0.9637	70.21	4.12%
TEG	0.9988	0.0002	0.88%	0.9411	0.0193	5.38%

**Fig. 12:**
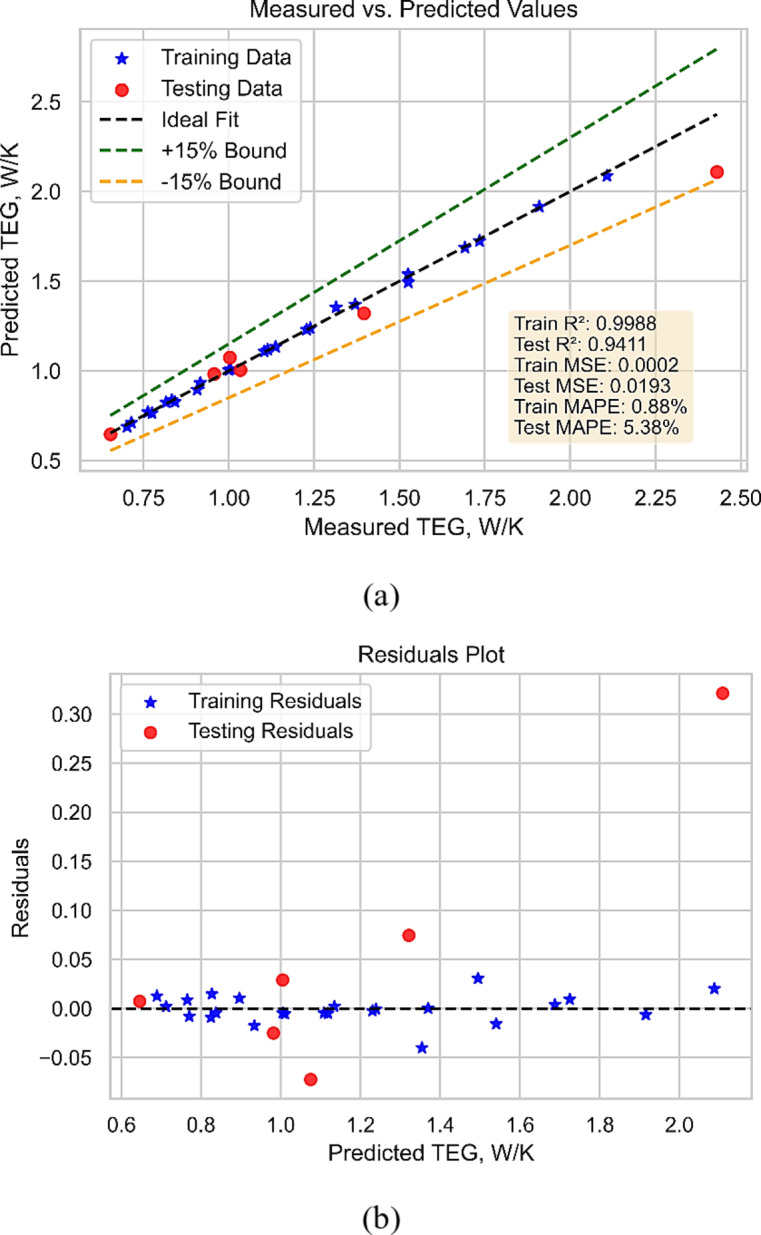
Total entropy generation model (**a**) performance (**b**) predicted vs. measured values.

Figure 12 depicts the performance of the GPR model in predicting TEG. Figure 12(a) depicts the predicted TEG plotted against the measured values, with the best-fit line depicted while Fig. 12(b) depicts the model’s residuals during predictions. The results show that the 5/2 Matern-based GPR model can accurately predict TEG for various operating situations and fluid parameters. Overall, the model performs well, as indicated by the good agreement between anticipated and measured TEG values. This highlights the usefulness of ML-based models for forecasting the behavior of complicated fluids like NFs and the significance of selecting a suitable kernel function for the GPR model.

With a smooth and flexible correlation structure, the performance criteria of the 5/2 Matérn GP) model show a rather accurate prediction capacity. The training MSE of 0.0002 and testing MSE of 0.0193 point to the model’s ability to preserve accuracy while fairly capturing complicated connections in the data. The model’s great capacity to provide consistent predictions is confirmed by the R² values of 0.9988 for training and 0.9411 for testing, which show that it properly compensates for the variation. Furthermore, underlining the great degree of accuracy of the model with little variance between predicted and real values are the MAPE values of 0.88% for training and 5.38% for testing. The test dataset’s somewhat greater error indicates that the model generalizes quite successfully across fresh data points—well within a reasonable range. The general consistency of these measures shows how well the 5/2 Matérn GPR model balances flexibility with smoothness in its forecasts. These findings confirm that the model is appropriate for uses needing exact function approximation, so it is a useful instrument for predictive modeling in many different fields of science and engineering.

### Correlations

The correlations are derived to estimate the friction factor and Nu of the HNFs based on the data acquired through testing presented in Eqs. (16) and (17).

For Nu,

16 $$\:\text{N}\text{u}\hspace{0.17em}=\hspace{0.17em}173.3\:\times\:\:{\text{R}\text{e}}^{0.7424}\times\:{\:\text{P}\text{r}}^{-4.555}{\times\:\left(1+\frac{{\upphi\:}}{100}\right)}^{55.83}$$

Average and standard deviation are 2.05 and 2.7%, respectively.

For friction factor,

17 $$\:{\text{f}\hspace{0.17em}=\hspace{0.17em}0.3281\:\times\:\:\text{R}\text{e}}^{-0.2549}\:\times\:{\left(1.0+\frac{{\upphi\:}}{100}\right)}^{10.98}$$

Average and standard deviation are 0.80 and 1.01%, respectively.

The above equations are valid for Reynolds’ number range of 7300 to 26,444, Prandtl number range of 5.3 to 5.5, and concentration range of 0.1 to 1.0 vol%, respectively.

## Conclusions

Numerical and experimental investigations were conducted to examine the hydrodynamic behavior and entropy generation of Al_2_O_3_-TiO_2_ (50:50) HNF flows in the tube under the CHF conditions at diverse Re. The data gathered from the lab-based analysis was used to create prognostic models using supervised machine learning techniques, specifically the 5/2 Matern Gaussian process regression. The findings of the study led to the following conclusions:

- The peak TC and VST change of 13.46 and 15.77% was observed for 1 vol% of HNF compared to the base fluid at 60 and 30^o^C, respectively.
- The peak improvement in HTC for HNF was noted for 1 vol%. concentration is 30.3% compared to Al_2_O_3_ NFs.
- The highest amplification in Nu for HNF and is 25.73%, respectively, at 1 vol% compared to Al_2_O_3_ NF.
- The peak $\:\varDelta\:p$ at 1 vol% concentration of HNF and Al_2_O_3_ NF is 21 and 13.65%, respectively, compared to water.
- The HNF’s friction factor was 8.02% greater in comparison to Al_2_O_3_ NF at 1 vol%.
- For HNF and Al_2_O_3_ NF at a concentration of 1 vol%, the highest TPF was 1.63 and 1.29, respectively.
- The generated models demonstrated good predictive effectiveness, with R^2^ values for friction factor as 0.9514, for Nu model as 0.9637, and for TEG model as 0.9411.
- The low modeling errors, ranging from 0 to 70.21, indicate a high level of accuracy in the predictions. These results demonstrate the robust predictive efficiency of the GPR model.

The present work provided valuable insights into the thermohydraulic performance of water-based nanofluids. Machine learning techniques are used to develop prognostic models for performance parameters. However, the researchers have the option to conduct experiments by dispersing the specified nanoparticles in high-temperature heat transfer fluids such as lubrication oils for diverse applications. There is a crucial need to delve deeper into the cost, stability, and nanoparticle concentration optimization aspects of nanofluids for long-term usage.

## Data Availability

The data is available within the manuscript and available upon reasonable request from the Corresponding author.

## Abbreviations

CFD: Computational fluid dynamics
GPR: Gaussian process regression
HNF: Hybrid nanofluid
HTF: Heat transfer fluid
ML: Machine learning
MSE: Mean squared error
NF: Nanofluid
NP: Nanoparticle
RBF: Radial basis function
SDBS: Sodium dodecylbenzene sulfonate
TC: Thermal conductivity, Wm^− 1^ K^− 1^
TEG: Total entropy generation, W K^− 1^
TPF: Thermal performance factor
VST: Viscosity, mPa.s

## List of symbols

A_s_: Test section surface area, m^2^
Be: Bejan number
c_p_: Specific heat, J kg^− 1^.K^− 1^
f: Friction factor
h: Heat transfer coefficient, W m^− 1^ K^− 2^
I: Current, amperes
*I*: Turbulent intensity
L: Tube length, m
m: Mass flow rate, kg s^− 1^
Pr: Prandtl number, $\text{Pr}=\frac{\mu {c}_{p }}{k}$
$\Delta \text{p}$: Pressure drop, Pa
Q: V $$\times$$ I, Watt
q: Heat flux, W m^− 2^
R: Correlation coefficient
R^2^: Coefficient of determination
Re: Reynolds number, $\text{Re}=\frac{\rho vD}{\mu }$
T_b_: Fluid bulk temperature, K
T_i_: Fluid inlet temperature, K
T_o_: Fluid outlet temperature, K
T_s_: Surface temperature, K
V: Volts, v
v: Velocity of the fluid, m s^− 1^

## Greek symbols

*µ*: Dynamic viscosity, mPa.s
*ρ*: Density, kg m^− 3^
*ϕ*: Volume concentration,

## Subscripts

*bf*: Base fluid
*hnf*: Hybrid nanofluid
*nf*: Nanofluid
*np*: Nanoparticle
*w*: Water
